# Estimation of CT-Derived Abdominal Visceral and Subcutaneous Adipose Tissue Depots from Anthropometry in Europeans, South Asians and African Caribbeans

**DOI:** 10.1371/journal.pone.0075085

**Published:** 2013-09-17

**Authors:** Sophie V. Eastwood, Therese Tillin, Andrew Wright, John Heasman, Joseph Willis, Ian F. Godsland, Nita Forouhi, Peter Whincup, Alun D. Hughes, Nishi Chaturvedi

**Affiliations:** 1 National Heart and Lung Institute, Imperial College London, London, United Kingdom; 2 Department of Medicine, Imperial College Healthcare NHS Trust, London, United Kingdom; 3 Department of Endocrinology and Metabolic Medicine, Imperial College London, London, United Kingdom; 4 MRC (Medical Research Council) Epidemiology Unit, University of Cambridge, Cambridge, United Kingdom; 5 Division of Population Health Sciences and Education, St. George’s University of London, London, United Kingdom; Charité University Medicine Berlin, Germany

## Abstract

**Background:**

South Asians and African Caribbeans experience more cardiometabolic disease than Europeans. Risk factors include visceral (VAT) and subcutaneous abdominal (SAT) adipose tissue, which vary with ethnicity and are difficult to quantify using anthropometry.

**Objective:**

We developed and cross-validated ethnicity and gender-specific equations using anthropometrics to predict VAT and SAT.

**Design:**

669 Europeans, 514 South Asians and 227 African Caribbeans (70±7 years) underwent anthropometric measurement and abdominal CT scanning. South Asian and African Caribbean participants were first-generation migrants living in London. Prediction equations were derived for CT-measured VAT and SAT using stepwise regression, then cross-validated by comparing actual and predicted means.

**Results:**

South Asians had more and African Caribbeans less VAT than Europeans. For basic VAT prediction equations (age and waist circumference), model fit was better in men (R^2^ range 0.59-0.71) than women (range 0.35-0.59). Expanded equations (+ weight, height, hip and thigh circumference) improved fit for South Asian and African Caribbean women (R^2^ 0.35 to 0.55, and 0.43 to 0.56 respectively). For basic SAT equations, R^2^ was 0.69-0.77, and for expanded equations it was 0.72-0.86. Cross-validation showed differences between actual and estimated VAT of <7%, and SAT of <8% in all groups, apart from VAT in South Asian women which disagreed by 16%.

**Conclusion:**

We provide ethnicity- and gender-specific VAT and SAT prediction equations, derived from a large tri-ethnic sample. Model fit was reasonable for SAT and VAT in men, while basic VAT models should be used cautiously in South Asian and African Caribbean women. These equations will aid studies of mechanisms of cardiometabolic disease in later life, where imaging data are not available.

## Introduction

Escalating global levels of obesity will fuel an epidemic of diabetes and cardiovascular disease [1]. Deposition of central adipose tissue, and in particular the relation between visceral and subcutaneous fat, contribute to the development of cardiometabolic disease, yet their respective roles remain incompletely understood [2–4]. There are marked ethnic differences in cardiometabolic risk, and in central adipose fat deposition [5,6]. People of South Asian (originating in the Indian subcontinent) and Black African origin share an excess risk of diabetes compared to people of European origin [7–9], but while South Asians also experience excess coronary heart disease (CHD) [10,11], African Caribbeans have lower rates than Europeans [12].

Surface anthropometry suggests that South Asians have excess visceral fat, while levels are low in African Caribbeans [6,13]. However, anthropometry cannot distinguish VAT and SAT, and correlations between anthropometrics and VAT or SAT differ between ethnic groups [5,6]. Especially in people of South Asian origin, measures such as body mass index (BMI) do not correlate well with VAT [14]. Therefore, direct measurement of fat distribution should improve prediction of cardiometabolic outcomes.

Both depots are accurately measured by computer tomography (CT) or magnetic resonance imaging (MRI) [15]. However, irradiation (with CT), expense and access difficulties prevent these techniques from being widely used. Consequently, VAT and SAT prediction equations, using anthropometrics as explanatory variables, have been generated from CT or MR imaging in European [16–20], North American [21–25] and Indian settings [26,27]. However, studies that have generated equations for South Asian or African Caribbean adult populations have either had small numbers of participants [21,26,27], excluded participants with chronic disease [22,26,27], excluded elderly participants [22,26], or do not provide equations for SAT estimation [22,27], These factors reduce their generalizability and utility. Additionally, not all of these studies internally cross-validated their equations [21,27], and none applied previous prediction equations to their datasets [21,22,26,27].

We therefore used CT and anthropometric data from a tri-ethnic (white European, South Asian and African Caribbean) population-based study to describe associations between fat depots and anthropometrics by gender and ethnicity, to develop and cross-validate gender- and ethnicity-specific VAT and SAT prediction equations and to explore the validity of previously published VAT prediction equations.

## Materials and Methods

### Ethics statement

All participants gave written informed consent. Approval for the follow-up study was obtained from St Mary’s Hospital Research Ethics Committee (ref. 07/H0712/109).

### Study sample

We used cross-sectional follow-up data from the Southall And Brent REvisited (SABRE) study, a community-based tri-ethnic cohort study of men and women living in north-west London. The cohort’s focus is on inter-ethnic differences in cardiovascular disease and diabetes, details have been published elsewhere [28]. All South Asian and African-Caribbean participants were first-generation migrants. Most African Caribbeans (92.5%) were born in the Caribbean and the remainder were born in West Africa. Most (82%) South Asians were born in the Indian subcontinent and 14% were born in East Africa, approximately half (52%) were of Punjabi Sikh origin. Ethnicity was confirmed by participants at interview. Participants aged 40-69 (n=4857) were randomly selected from age- and gender-stratified general practitioner lists and workplaces at baseline (1988-1991), and were followed-up between 2008 and 2011, aged 58 to 85 years (n=4196). A total of 1410 people attended a research clinic at follow-up (2008-11) and underwent CT scanning. Of these, 669 were white European (23% female), 514 were South Asian (15% female) and 227 were African-Caribbean (49% female), (Figure S1, supporting information). As the baseline study had been initially designed to study ethnic differences in cardiometabolic disease in men, a male preponderance in the data exists. This is absent in African Caribbeans, who were recruited later into the study, when the importance of cardiovascular disease in women was better recognised.

### Anthropometric measurements

At the follow-up visit, measurements were undertaken by trained researchers in the study clinic at St Mary’s Hospital, London. Height was measured using a stadiometer, with the participant barefoot and standing straight with the head level. Participants were weighed barefoot wearing a hospital gown, using a Tanita TBF-410 MA body composition analyser. This was used to calculate fat % and fat mass (in kg), in addition to total weight. Waist circumference was measured halfway between the costal margin and the iliac crest. Hip circumference was measured at the level of the greater trochanter. Thigh circumference was measured by instructing the participant to place their right foot on a chair, identifying and marking the midpoint between the hip crease and the patella, instructing the participant to return their leg to a straight position, and then measuring the circumference at the marked point. Circumferences were measured to the nearest millimetre using a fibre glass tape with a spring balance set to a constant tension of 600g. Body mass index (BMI) was calculated as weight in kg/ (height in m)^2^. We assessed the reliability of anthropometric measurements by comparing two sets of measurements from one observer on a sample of thirty participants. The coefficients of variation for weight, height and waist, hip and thigh circumferences were 0.16%, 0.23%, 1.98%, 0.71% and 7.50% respectively.

### Radiological measurements

Visceral and subcutaneous adipose tissue (VAT and SAT) were measured by computer tomography (CT) scan at 125kV with a Philips MX 8000 IDT64 detector, which scanned a single slice of 10mm thickness at the fourth lumbar (L4) vertebral level. On average, the largest proportion of fat is seen at L4, most authors claim VAT at L4-5 level is best correlated with total VAT volume, and scans at this level best allow differentiation between VAT and SAT [29]. Images were taken with the participant supine, in fixed inspiration and with their arms extended overhead.

**Figure 1:**
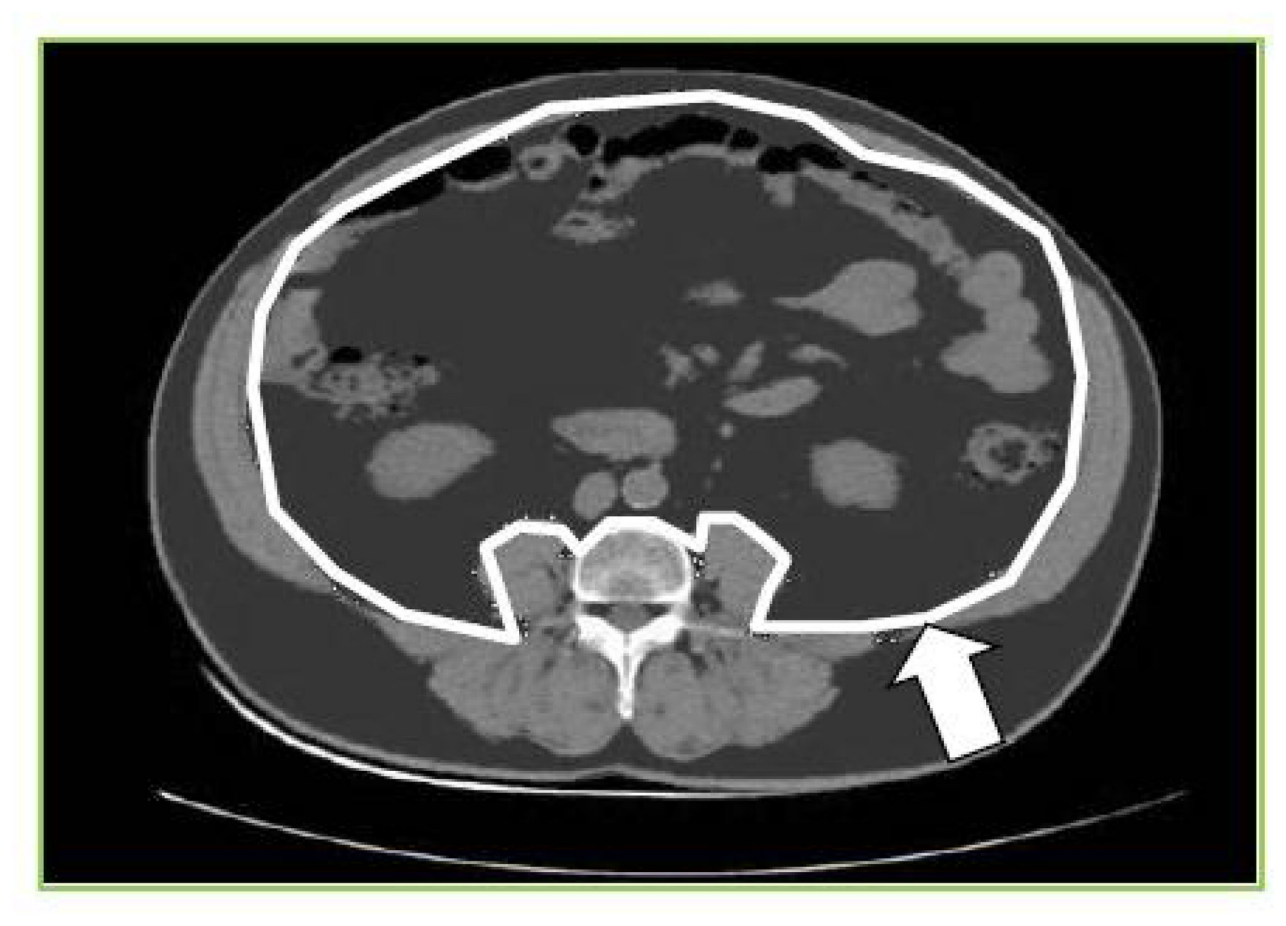
Measurement of VAT and SAT from CT image at L4. Adipose tissue is dark grey, white line shows delineation of visceral compartment, white arrow indicates depth of subcutaneous compartment.

VAT was measured on the CT image by circumscribing the visceral compartment manually (using Image-J software [30]) and using an attenuation range of -190 to -30 Hounsfield units to quantify adipose tissue within. Total abdominal adipose tissue was measured using an automated function that gave the adipose tissue in the whole cross-sectional slice. SAT was calculated by subtracting the area of adipose tissue within outer border of the abdominal wall musculature from the total adipose tissue, using the above attenuation range (Figure 1).

The reliability of VAT and SAT measurements was tested by comparing two sets of measurements from one observer on a sample of thirty participants. The coefficient of variation for VAT was 0.95% and for SAT 0.68%.

### Statistical analyses

Data were split randomly within gender and ethnic group, creating a derivation data sub-set (~ 66%), and a validation data sub-set (~ 33%). Summary statistics were generated by gender, ethnicity and derivation/ validation sub-set. ANOVA or chi-squared tests (for continuous/ categorical data respectively) were used to determine ethnic differences and differences between derivation and validation data sub-sets. Spearman’s correlation coefficients were calculated for associations between VAT or SAT and age and anthropometrics in the derivation sub-set, by gender and ethnicity.

Two strategies were used to create prediction equations. Firstly, models were generated for VAT using age and waist circumference, and for SAT using age, weight and height. These models aimed to provide prediction tools when only basic anthropometry is available, as is likely to be the case in most research studies. Secondly, backwards stepwise regression was used with age (as a forced variable) and the following in the initial model; height, weight, waist, hip and thigh circumferences. Variables were eliminated sequentially until all variables in the model were significant at p<0.2. This process was carried out for each gender and ethnic group within the derivation dataset. We chose to represent weight, height, waist and hip circumferences as separate variables in models, rather than using body mass index (BMI) or waist:hip ratio. This was to better appreciate effects of each parameter on VAT/ SAT and to avoid multicollinearity in models. Age was specified a priori as a forced variable in all models as body fat distribution is thought to vary with age, with preferential VAT deposition with increasing age, particularly in women [31]. Goodness of fit was assessed with the adjusted R^2^ statistic.

VAT and SAT prediction equations were internally cross-validated, using the validation dataset to compare actual mean VAT or SAT values with mean VAT or SAT predicted by equations, and expressing these as proportions of the respective actual means. The difference was plotted for each group to enable visual inspection of model fit. Additionally, prediction equations from studies that used similar anthropometrics as predictors were applied to the validation dataset (for South Asian and African Caribbean participants separately, and then to the whole validation data subset) and differences between resulting actual and predicted mean VAT/ SAT values were compared [17,22,24,26,27,32] (table S1, supporting information).

Sensitivity analyses were performed (comparing R^2^ values) on the basic models described for VAT and SAT (above), under the following conditions: the addition of total fat mass/ percentage values (from bioimpedance data)/ body mass index, participants with and without diagnosed diabetes at follow-up (defined as a diagnosis of diabetes/ prescription of anti-diabetic medications from primary care record review, or recall of physician-diagnosed diabetes plus either year of diagnosis or receipt of named anti-diabetic medication from participant questionnaire, or fasting/ oral glucose tolerance test plasma glucose results meeting WHO 1999 criteria at 2008-2011 follow-up [33]), and participants in the younger (56-72 years) and older (73-86 years) age groups.

All analyses were performed using Stata 12 (College Station, Texas).

## Results

White European men were generally larger in all anthropometric and body composition parameters compared with South Asian men (table 1). However, after adjusting for age, height and total fat mass, South Asian men had the greatest, and African Caribbean men the smallest areas of visceral adipose tissue (VAT). South Asian men also had the greatest area of subcutaneous adipose tissue (SAT), followed by African Caribbean men, then white European men. African Caribbean women were the heaviest, with the largest waist, hip and thigh measurements, whereas South Asian women generally had the smallest measurements (table 2). Reflecting the trend in men, South Asian women had the greatest adjusted VAT and SAT areas, with African Caribbean women having the lowest VAT area, and white European women having the lowest SAT area. In both genders, there was evidence for inter-ethnic differences for most anthropometrics. Characteristics did not differ between the derivation and validation sub-sets in either gender (all p-values >0.05).

**Table 1:** Characteristics of men (n=1071) in the derivation and validation datasets – SABRE study 2008-2011.

	**Derivation dataset, n=727**				**Validation dataset, n=344**			
**Independent variables**	**White European**	**South Asian**	**African Caribbean**	**p inter-ethnic difference**	**White European**	**South Asian**	**African Caribbean**		**p inter-ethnic difference**
n	349	300	78	-	168	139		37	-
Age, years	70±6	69±6	71±6	0.01	70±6	69±7		71±5	0.08
Current smoking, %	7	4	5	<0.001	11	6		8	<0.001
Diabetes, %	13	39	37	<0.001	19	31		27	0.04
Systolic blood pressure, mmHg	140±17	142±18	146±17	0.03	137±16	143±18		142±15	0.007
On β-blockers, %	19	30	10	<0.001	18	27		22	0.14
Total cholesterol, mmol/l	4.8±1.1	4.4±1.0	4.5±1.1	<0.0001	4.7±1.1	4.4±1.0		4.8±1.1	0.05
Lipid-lowering medication, %	52	70	59	<0.001	51	68		49	0.008
Height, cm	173±7	169±6	171±7	<0.0001	174±7	168±6		171±7	<0.0001
Weight, kg	83±14	75±12	81±12	<0.0001	84±15	74±11		82±13	<0.0001
BMI, kg/m²	28±4	26±4	28±4	<0.0001	28±5	26±3		28±4	0.0004
Waist circumference, cm	101±13	99±10	98±12	0.002	102±13	98±10		99±11	0.004
Hip circumference, cm	103±9	97±8	98±7	<0.0001	103±9	98±7		99±9	<0.0001
Thigh circumference, cm	51±4	49±5	55±8	<0.0001	52±6	50±6		54±5	0.0004
Waist:hip ratio	0.99±0.06	1.01±0.06	0.99±0.07	<0.0001	0.99±0.06	1.00±0.06		1.00±0.05	0.22
Total body fat mass, kg	23±9	20±8	21±8	<0.0001	24±10	19±7		22±7	<0.0001
Total body fat %	27±6	26±6	25±6	0.02	27±7	25±6		26±5	0.0204
VAT area, cm²	241±99	234±91	191±102	0.0002	253±114	216±84		198±71	0.0006
Adjusted^1^ VAT area, cm²	227±69	248±70	192±67	0.0002	236±74	235±76		196±72	0.0005
SAT area, cm²	224±89	229±84	209±91	0.19	219±90	226±85		237±95	0.48
Adjusted^1^ SAT area, cm²	209±50	243±50	213±48	<0.0001	202±56	244±57		230±54	<0.0001

Data are given as mean ± SD unless otherwise stated, ^1^adjusted for age, height and total fat mass.

**Table 2:** Characteristics of women (n=339) in the derivation and validation datasets: SABRE study 2008-2011.

		**Derivation dataset, n=229**					**Validation dataset, n=110**			
**Independent variables**	**White European**		**South Asian**	**African Caribbean**	**p inter-ethnic difference**	**White European**		**South Asian**	**African Caribbean**	**p inter-ethnic difference**
n	99		57	73	-	53		18	39	-
Age, years	70±6		68±6	69±6	0.37	69±6		67±6	71±6	0.08
Current smoking, %	7		4	5	<0.001	9		6	5	0.001
Diabetes, %	14		25	32	0.028	13		22	49	0.001
Systolic blood pressure, mmHg	135±18		140±17	138±18	0.21	134±16		145±26	141±18	0.04
On β-blockers, %	15		25	15	0.27	15		17	15	0.99
Total cholesterol, mmol/l	5.4±1.1		4.9±1.3	4.8±1.1	0.004	5.4±1.1		5.1±1.2	5.0±1.1	0.23
Lipid-lowering medication, %	38		67	38	0.001	36		56	67	0.012
Height, cm	160±6		153±6	160±5	<0.0001	162±7		153±6	158±6	<0.0001
Weight, kg	71±14		65±12	79±14	<0.0001	75±18		66±14	75±16	0.15
BMI, kg/m²	28±5		28±5	31±6	0.0005	29±6		29±5	30±7	0.42
Waist circumference, cm	93±13		96±11	99±13	0.02	96±15		96±11	97±14	0.87
Hip circumference, cm	103±10		102±10	107±11	0.008	105±11		102±11	105±12	0.58
Thigh circumference, cm	54±7		53±7	59±7	<0.0001	55±8		56±10	56±6	0.95
Waist:hip ratio	0.90±0.07		0.95±0.09	0.92±0.07	0.001	0.91±0.09		0.95±0.08	0.93±0.07	0.17
Total body fat mass, kg	27±9		25±8	31±9	0.0003	30±13		26±10	30±11	0.35
Total body fat %	37±7		37±7	39±6	0.05	38±8		38±7	39±7	0.85
VAT area, cm²	153±77		156±62	141±62	0.38	160±73		181±74	161±92	0.60
Adjusted^1^ VAT area, cm²	155±56		170±61	125±57	<0.0001	164±8		188±14	152±9	0.09
SAT area, cm²	278±112		317±119	355±134	0.0003	315±129		317±93	352±139	0.37
Adjusted^1^ SAT area, cm²	291±61		331±66	312±62	0.0004	317±65		327±69	344±64	0.15

Data are given as mean ± SD unless otherwise stated, ^1^adjusted for age, height and total fat mass.

In univariate analysis, age and height were not consistently associated with VAT or SAT. Weight, BMI, waist:hip ratio and waist, hip and thigh circumferences were generally positively associated with VAT and SAT in both genders and all ethnic groups, with the strongest associations tending to be with waist circumference for VAT and weight or BMI for SAT. For VAT, anthropometric associations were mostly weakest in South Asians (table 3).

**Table 3:** Univariate associations between VAT and SAT and independent variables, by gender and ethnicity - SABRE study 2008-2011.

	**Men**						**Women**					
	**White European**		**South Asian**		**African Caribbean**		**White European**		**South Asian**		**African Caribbean**	
	**n=344**		**n=299**		**n=78**		**n=99**		**n=57**		**n=70**	
**Independent variables**	**rho^1^**	**p**	**rho^1^**	**p**	**rho^1^**	**p**	**rho^1^**	**p**	**rho^1^**	**p**	**rho^1^**	**p**
**VAT**												
Age, years	0.09	0.10	0.05	0.38	0.33	0.004	0.08	0.42	0.17	0.20	0.11	0.36
Height, cm	0.01	0.83	-0.004	0.95	-0.14	0.23	0.20	0.05	-0.11	0.40	-0.04	0.75
Weight, kg	0.63	<0.0001	0.59	<0.0001	0.60	<0.0001	0.73	<0.0001	0.39	0.003	0.50	<0.0001
BMI, kg/m²	0.69	<0.0001	0.69	<0.0001	0.75	<0.0001	0.68	<0.0001	0.47	0.0002	0.55	<0.0001
Waist circumference, cm	0.77	<0.0001	0.77	<0.0001	0.85	<0.0001	0.75	<0.0001	0.57	<0.0001	0.66	<0.0001
Hip circumference, cm	0.57	<0.0001	0.56	<0.0001	0.57	<0.0001	0.65	<0.0001	0.21	0.12	0.36	0.002
Thigh circumference, cm	0.41	<0.0001	0.34	<0.0001	0.49	<0.0001	0.47	0.0001	0.02	0.89	0.10	0.42
Waist:hip ratio	0.72	<0.0001	0.61	<0.0001	0.77	<0.0001	0.54	<0.0001	0.52	<0.0001	0.64	<0.0001
Total body fat mass, kg	0.69	<0.0001	0.68	<0.0001	0.78	<0.0001	0.73	<0.0001	0.37	0.005	0.50	<0.0001
Total body fat %	0.64	<0.0001	0.66	<0.0001	0.78	<0.0001	0.67	<0.0001	0.36	0.01	0.39	0.0007
**SAT**												
Age, years	-0.10	0.06	-0.06	0.31	0.27	0.02	-0.03	0.74	-0.24	0.07	-0.06	0.62
Height, cm	0.11	0.05	0.06	0.30	-0.01	0.96	0.09	0.35	-0.10	0.48	0.03	0.78
Weight, kg	0.78	<0.0001	0.72	<0.0001	0.72	<0.0001	0.78	<0.0001	0.80	<0.0001	0.80	<0.0001
BMI, kg/m²	0.78	<0.0001	0.80	<0.0001	0.78	<0.0001	0.80	<0.0001	0.86	<0.0001	0.83	<0.0001
Waist circumference, cm	0.80	<0.0001	0.79	<0.0001	0.80	<0.0001	0.74	<0.0001	0.66	<0.0001	0.77	<0.0001
Hip circumference, cm	0.79	<0.0001	0.71	<0.0001	0.76	<0.0001	0.86	<0.0001	0.83	<0.0001	0.89	<0.0001
Thigh circumference, cm	0.66	<0.0001	0.59	<0.0001	0.58	<0.0001	0.63	<0.0001	0.71	<0.0001	0.63	<0.0001
Waist:hip ratio	0.51	<0.0001	0.43	<0.0001	0.54	<0.0001	0.30	0.003	-0.04	0.74	0.17	0.16
Total body fat mass, kg	0.81	<0.0001	0.80	<0.0001	0.81	<0.0001	0.79	<0.0001	0.83	<0.0001	0.80	<0.0001
Total body fat %	0.73	<0.0001	0.74	<0.0001	0.75	<0.0001	0.70	<0.0001	0.77	<0.0001	0.66	<0.0001

rho**^1^**=Spearman correlation coefficients, results from derivation dataset only.

### Prediction models

Waist circumference was used in VAT basic models, and height and weight in SAT basic models, as waist and BMI were the simple measurements most correlated with VAT or SAT respectively in most sex/ ethnic groups, table 3. We used height and weight in SAT models rather than BMI in order to better appreciate the relative contribution of each measure. Basic prediction models for SAT (age, weight and height: adjusted R^2^ 0.69-0.77) fitted better than those for VAT (age and waist: adjusted R^2^ 0.35-0.71). Model fit for VAT was better in men (adjusted R^2^ range 0.59-0.71) than in women (adjusted R^2^ range 0.35-0.59). Conversely, basic SAT models fitted better in women (adjusted R^2^ range 0.75-0.77) than men (adjusted R^2^ range 0.69-0.72) (table 4 for equations and table S2 of supporting information for full models).

**Table 4:** Prediction equations for VAT and SAT, by gender and ethnicity - SABRE study 2008-2011.

**Gender/ ethnic group**	**Model^1^**	**Predictive equation**	**Adjusted R^2^**
***VATincm*** ^2^			
White European men	Basic	-554.67 + (1.88 x age) + (6.57 x waist)	0.64
	Expanded	67.88 + (1.87 x age) + (2.80 x weight) – (1.79 x height) + (7.57 x waist) – (4.91 x hip)	0.69
		- (2.85 x thigh)	
South Asian men	Basic	-523.36 + (1.44 x age) + (6.67 x waist)	0.59
	Expanded	39.24 + (1.18 x age) + (2.16 x weight) – (2.35 x height) + (6.56 x waist) – (2.15 x hip)	0.61
		- (1.81 x thigh)	
African Caribbean men	Basic	-598.50 + (1.14 x age) + (7.24 x waist)	0.71
	Expanded	-422.31 + (0.94 x age) + (8.63 x waist) - (3.86 x hip) + (1.47 x thigh)	0.73
White European women	Basic	-328.49 + (0.68 x age) + (4.66 x waist)	0.59
	Expanded	-250.86 + (0.90 x age) + (2.71 x weight) + (2.62 x waist) – (1.75 x thigh)	0.62
South Asian women	Basic	-276.47 + (1.76 x age) + (3.27 x waist)	0.35
	Expanded	475.69 + (1.13 x age) + (6.28 x weight) – (2.47 x height) + (1.85 x waist) - (3.71 x hips) – (4.35 x thigh)	0.55
African Caribbean women	Basic	-294.41 + (1.82 x age) + (3.15 x waist)	0.43
	Expanded	323.39 +(2.20 x age) + (3.20 x weight) – (2.42 x height) + (3.35 x waist) – (4.93 x hip)	0.56
***SATincm*** ^2^			
White European men	Basic	290.49 - (0.34 x age) + (5.67 x weight) – (3.00 x height)	0.69
	Expanded	-28.95 – (1.19 x age) + (2.29 x weight) – (2.00 x height) + (2.34 x waist) + (2.49 x hip)	0.72
South Asian men	Basic	418.22 + (1.00 x age) + (6.72 x weight) – (4.51 x height)	0.72
	Expanded	48.58 + (0.63 x age) + (3.88 x weight) – (3.21 x height) + (1.43 x waist) + (2.54 x hip)	0.75
African Caribbean men	Basic	473.94 + (1.73 x age) + (6.49 x weight) – (5.34 x height)	0.72
	Expanded	-373.61 – (0.08 x age) – (2.09 x height) + (3.83 x waist) + (6.52 x hip) – (1.23 x thigh)	0.81
White European women	Basic	461.22 - (0.59 x age) + (7.22 x weight) – (4.09 x height)	0.75
	Expanded	24.98 – (0.99 x age) + (3.76 x weight) – (3.07 x height) + (7.01 x hip) – (3.03 x thigh)	0.80
South Asian women	Basic	1071.90 - (2.59 x age) + (8.52 x weight) – (7.39 x height)	0.77
	Expanded	404.25 – (1.72 x age) + (3.94 x weight) – (5.21 x height) + (5.63 x hip)	0.81
African Caribbean women	Basic	619.42 + (1.51 x age) + (8.54 x weight) – (6.50 x height)	0.76
	Expanded	-896.27 + (0.23 x age) + (2.24 x waist) + (9.48 x hip)	0.86

^1^Basic model used the following predictors: VAT; age (years), waist circumference (cm) and SAT; age (years), height (cm), weight (kg)

^1^Expanded model used the following predictors for both depots: age (years), weight (kg), height (cm), waist circumference (cm), hip circumference (cm), thigh circumference (cm).

Expanded models were constructed with age as a forced variable and height, weight, waist, hip and thigh circumferences as potential factors in a backwards stepwise regression. For VAT and SAT, expanded models improved adjusted R^2^ when compared to basic models, especially in South Asian and African Caribbean women for VAT and African Caribbeans of both genders for SAT (table 4, table S2).

**Figure 2:**
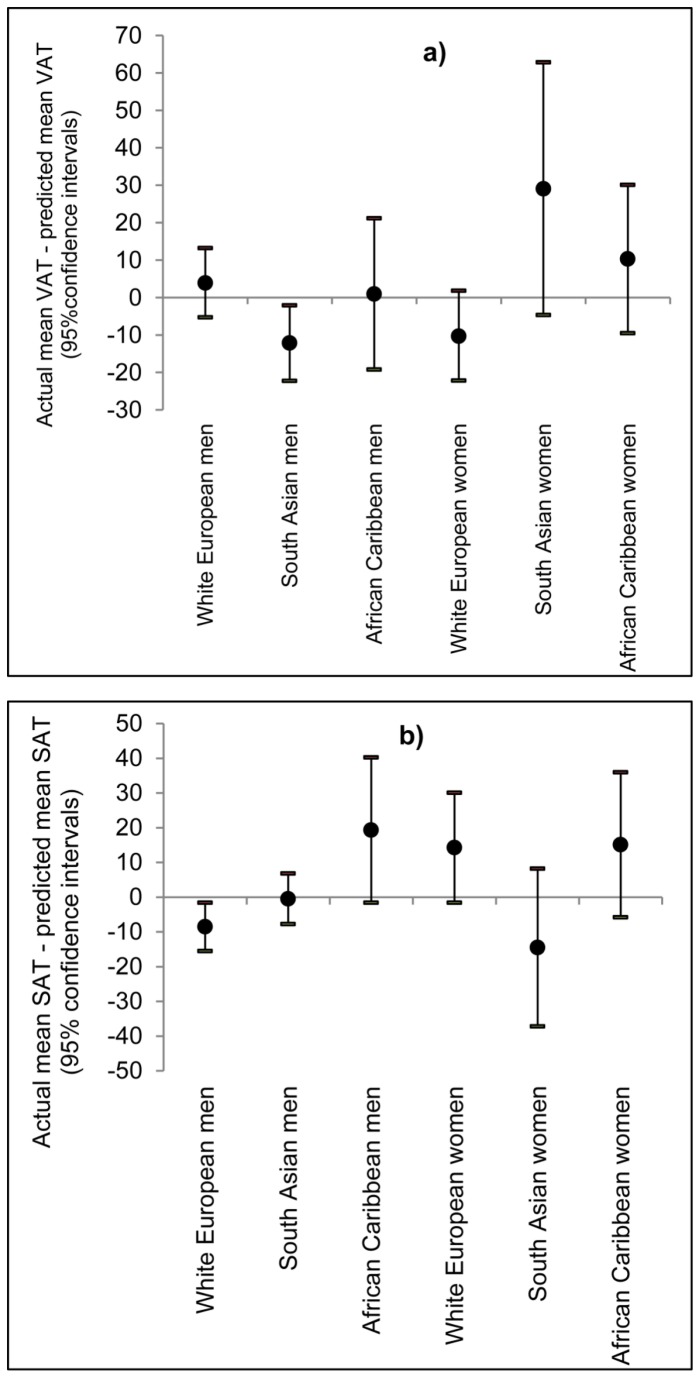
Cross-validation: differences between actual and predicted mean for a) VAT and b) SAT. Cross-validation was performed in the validation dataset, by gender and ethnicity, using equations using age, height, weight, and waist / hip / thigh circumferences as predictors.

Basic and expanded models were cross-validated by comparison of mean actual and mean predicted values (for VAT and SAT) on the validation dataset, by gender and ethnicity. For VAT and SAT, differences between predicted and actual means were generally small, ranging from 1.2% to 9.3% of actual means for basic VAT models, 0.5% to 6.7% for expanded VAT models, 0.3% to 9.5% for basic SAT models and 0.2% to 8.1% for expanded SAT models. The exceptions to this were VAT models for South Asian women, which showed an 11.8% difference for the basic model and a 16.0% difference for the expanded model (see Figure 2).

**Figure 3:**
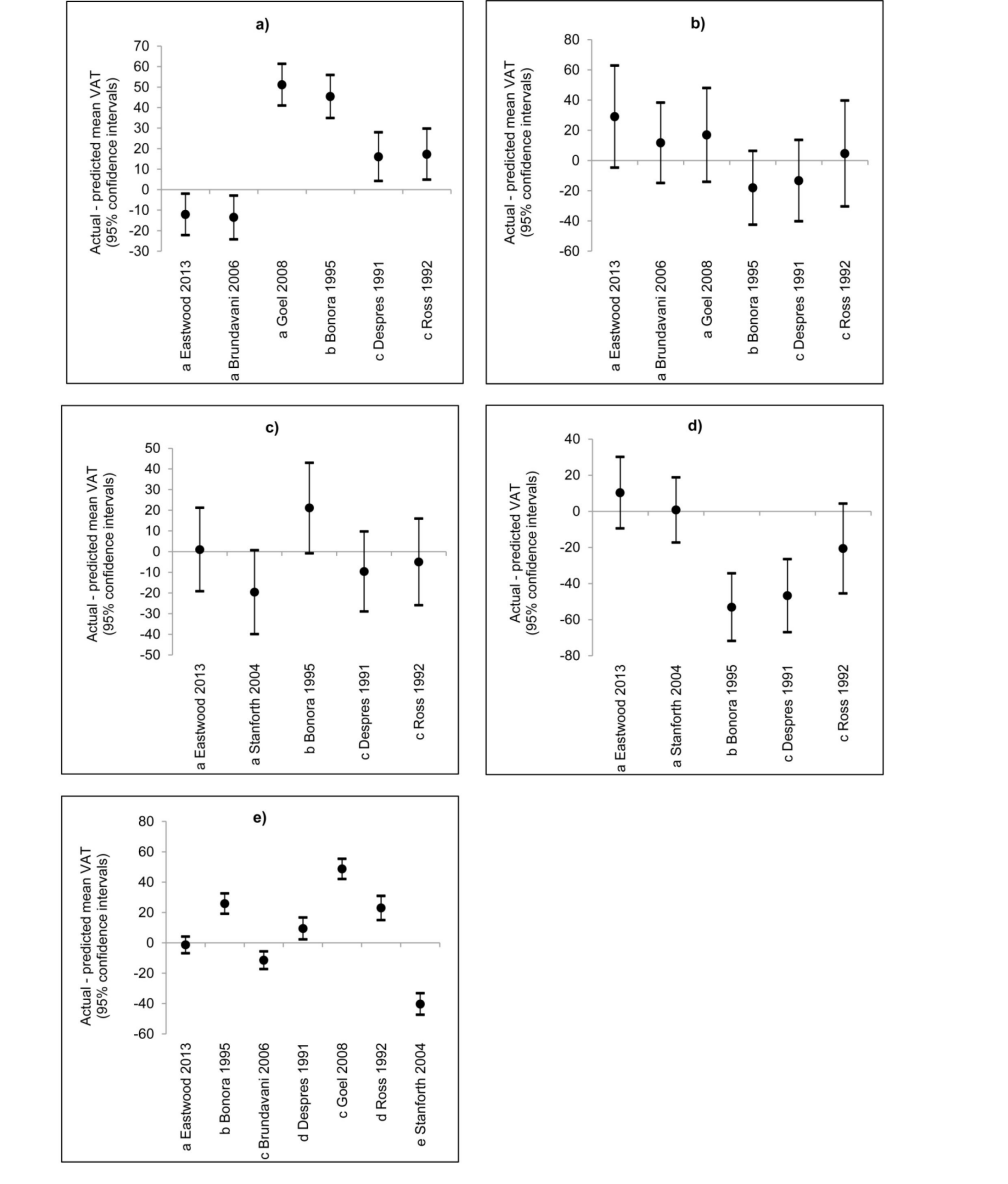
Inter-study differences between actual and predicted mean VAT in: a) South Asian men, b) South Asian women, c) African Caribbean men, d) African Caribbean women and e) men and women of all ethnicities. Inter-study VAT cross-validation was performed in the validation dataset. Results from this study show predicted means from equations using age, height, weight, waist / hip/ thigh circumferences as predictors. For Figure 3a) and b). ^a^ Equations derived from male and female populations of South Asian origin. ^b^ Equations derived from male and female populations of white European origin. ^c^ Equations derived from male populations of white European origin. For Figure 3c) and d). ^a^ Equations derived from male and female populations of African Caribbean/ American origin. ^b^ Equations derived from male and female populations of white European origin. ^c^ Equations derived from male populations of white European origin. For Figure 3e):. ^a^ Equations derived from male and female populations of white European, South Asian and African Caribbean origin. ^b^ Equations derived from male and female populations of white European origin ^c^ Equations derived from male and female populations of South Asian origin ^d^ Equations derived from male populations of white European origin ^e^ Equations derived from male and female populations of white European and African American origin

VAT prediction equations from previous studies were validated separately in our South Asian and African Caribbean populations, then in the whole validation data subset (comparing two equations derived from Indian populations living in India [26] [27], one from an African American population [22], and three from white European Canadian/North American populations [17,24,32]. Only equations using similar anthropometrics (see table S1, supporting information for external equations in full) were compared to the expanded equations described in this study, using the validation dataset, see Figure 3. In South Asians, Brundavani et al’s equations performed similarly to ours for men and women (i.e. difference in actual and predicted means as a proportion of the actual mean did not differ from that found in our cross-validation by >10%) [27]. Goel et al’s equations did not perform as well for men (difference in means: Goel et al; 23.6% of actual mean, our equations; 5.6%) [26]. For African Caribbeans, the equations of Stanforth et al worked similarly to our equations in both genders [22]. Our equations performed better than those of Despres et al, Bonora et al and Ross et al in South Asian men and African Caribbeans of both sexes, though not in South Asian women [17,24,32]. When equations were applied across all participants in the validation dataset, our equations performed better than all externally-derived ones (difference in means: our equations; 0.6% of actual mean, externally-derived equations; 4.4% (Despres et al) to 22.8% (Goel et al)) [17,22,24,26,27,32].

Sensitivity analyses adding fat mass or BMI to models or deriving models in subgroups (people with diabetes or age < 73) did not generally show large differences in model adjusted R^2^ values, and none that were replicated consistently across gender and ethnic groups (table S3 supporting information).

## Discussion

In this large UK cross-sectional population-based study we provide novel and validated gender- and ethnic-specific equations for estimating abdominal visceral and subcutaneous fat deposition from anthropometric data. VAT has been consistently linked with conventional cardiovascular risk factors [34], emergent risk factors [35] and type 2 diabetes [36]. However, contention exists over whether SAT has a neutral, protective or detrimental role, as well as possible explanations for this [3,4]. Ethnic differences exist in fat deposition, with South Asian groups having more VAT and African Caribbeans more SAT, relative to white Europeans - these differences have been linked to differing cardiometabolic disease rates [6,37]. Therefore, inter-ethnic variation in patterns between fat deposition and cardiometabolic disease can help understanding of aetiology, identify mechanisms of disease and highlight pathways for intervention. Anthropometric measurements alone cannot distinguish between visceral and subcutaneous fat deposition, and are unreliable indicators of fat deposition in some ethnic groups [6,21]. Consequently, accurate quantification of VAT and SAT deposition is important to inform future research in this field.

We showed expected ethnic and gender differentials in CT-quantified VAT and SAT i.e. greater VAT in men than women, greater SAT in women than men, greater VAT and SAT in South Asians than white Europeans and lower VAT and more SAT in African Caribbeans than white Europeans [5,22,38,39]. Univariate associations between fat depots and anthropometrics were generally weakest in South Asians, reflecting low correlations found in previous work [26].

Whilst variance explained by the prediction equations differed by fat depot, gender and ethnicity and use of simple or expanded models, goodness of fit for VAT prediction equations was similar to that found in previous studies, with authors reporting R^2^ values of 0.51 [21] to 0.81 [32] for comparable models. Corresponding with the results from this study, previous work has found that VAT models’ goodness of fit was low in South Asians [26], and in women of all ethnic groups studied [18], [22], [27]. Other studies have reported high R^2^ values for SAT prediction models (0.67-0.85) [16,18,25,26], in keeping with this study, and where reported, these have been higher for women.

Cross-validation of our models, by comparison of actual and predicted means, suggested VAT models performed well in all groups except for South Asian women. However, for basic equations, the difference in the means was 21.4 cm^2^, or 11.8% of the actual mean, which compares favourably with results from equations derived by Goel et al, who reported a difference in means of 18.6 cm^2^ (15.8% of the actual mean), and Bonora et al, who reported a difference in means of 36.0 cm^2^ (21.3% of the actual mean) [17,26]. Differences between the true and estimated mean values of both depots were not numerically large, and were similar or an improvement on those reported by other studies. Where possible, we applied other authors’ VAT prediction equations to our validation dataset. Equations derived from South Asian populations and the equation derived from an African American population performed similarly to our equations in these ethnic groups, with the exception of Goel et al’s equations, which performed less well in South Asian men - possibly due to smaller numbers (n=95 for men) and a younger age range (18-50 years) of participants used to derive their equations [22,26,27]. As expected given their derivation from the same sample, our equations performed markedly better than any of the other equations tested when applied to the entire validation dataset [17,22,24,26,27,32]. Nevertheless, the comparison demonstrates the importance of gender- and ethnicity-specific equations in improving accuracy of VAT prediction at a population level.

This study has a number of strengths. To our knowledge, ours is the largest study to derive VAT prediction equations (n=1410), with only 2 other studies exceeding 200 participants (Brambilla et al: n=407, Stanforth et al: n=692) [20,22]. This is the first study to provide group-specific equations, rather than to include ethnicity as an adjustment factor in equations, which enables between ethnic group comparisons. We derived equations from easily and commonly measured anthropometrics; many other studies have presented equations derived from sagittal diameters or skinfold thickness [21,24,25] which may not be available in the majority of available datasets. Advantages of the methods employed include use of “generous” p-values for inclusion into complex models (P<0.2), to make use of effects which may not have high levels of statistical significance but may contribute importantly to VAT or SAT variation. We cross-validated our equations, and those of other studies, using a validation sub-set of the sample. Several previous studies have internally cross-validated their equations [17,19,20,22], but as far as we are aware, only 2 have compared them to equations derived from other studies [25,32] – both of which contained relatively small numbers of white male participants only.

Although the sample was population-based, caution is needed when generalising results to different ethnic groups, i.e. most South Asians in this study were of Indian origin, therefore results may not be generalisable to other groups originating from the Indian subcontinent, e.g. Bangladeshis or Sri Lankans. For the basic VAT models, goodness of fit was poor for South Asian and African Caribbean women (R^2^ 0.35 and 0.43 respectively). Therefore it is recommended that the more expanded equations be used, if possible, in these groups. This study pertains to individuals aged only 56 and above, therefore the pre-menopausal hormonal influence on adipose tissue deposition in women is not accounted for in prediction equations [40]. However, since cardiometabolic disease is more common in mid to later life, and many population studies focus on this age group, we do not consider this to be a major drawback of this work. Maislin et al have recently suggested VAT quantification at L2-3, rather than L4-5, more accurately reflects total VAT volume [41]. However their findings were from an obese white European population and are unlikely to apply to our population. Errors in anthropometric measurements may have affected the reliability of our prediction equations. However, as the coefficients of variation for repeated anthropometric measurements on a sub-sample were low, we do not believe measurement inaccuracies will have substantially affected the equations’ reliability.

In summary, this is the first UK-based population study to present ethnicity and gender-specific VAT and SAT prediction equations using routine anthropometrics in Europeans, South Asians and African Caribbeans. Model fit was reasonable for all ethnic groups for SAT and for VAT in men, while basic VAT models should be used cautiously in South Asian and African Caribbean women. Where imaging data are not available, these equations can aid studies of mechanisms of cardiometabolic disease in all ethnic groups.

## Supporting Information

Figure S1 **Flow through the SABRE study.**
(TIF)

Table S1 **VAT prediction equations for populations of South Asian and African origin from previous studies (17, 22, 24, 26, 27, 31).**
(DOCX)

Table S2 **Full prediction models for VAT and SAT basic and expanded models, SABRE study 2008-2011.** ^1^Basic models used the following predictors: VAT: age (years), waist circumference (cm) and SAT: age (years), height (cm), weight (kg). ^2^Expanded model used the following predictors for both depots: age (years), weight (kg), height (cm), waist circumference (cm), hip circumference (cm), thigh circumference (cm).
(DOCX)

Table S3 **Sensitivity analyses of VAT and SAT prediction models: effects on adjusted R^2^.** Empty cells indicate when terms did not stay in the model after backwards stepwise regression.
(DOCX)
