# Supplementary figures and images for: Estimation of CT-Derived Abdominal Visceral and Subcutaneous Adipose Tissue Depots from Anthropometry in Europeans, South Asians and African Caribbeans
Source: PLoS One. 2013 Sep 17;8(9):e75085. doi: 10.1371/journal.pone.0075085 (PMC3775834; doi:10.1371/journal.pone.0075085)

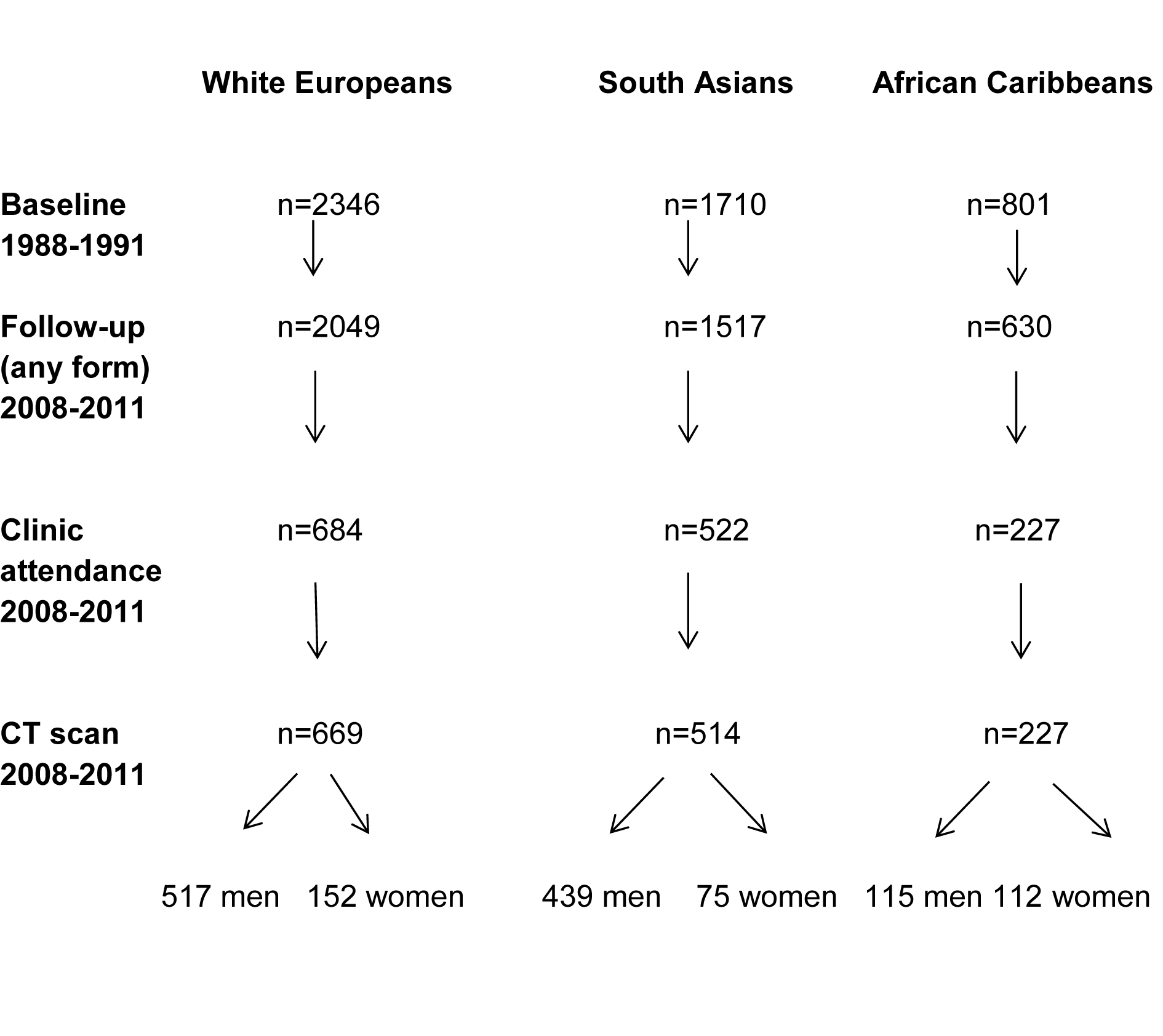

Supplement: Figure S1 — Flow through the SABRE study. (TIF) [file pone.0075085.s001.tif]
