# Supplementary material for: Estimation of CT-Derived Abdominal Visceral and Subcutaneous Adipose Tissue Depots from Anthropometry in Europeans, South Asians and African Caribbeans
Source: PLoS One. 2013 Sep 17;8(9):e75085. doi: 10.1371/journal.pone.0075085 (PMC3775834; doi:10.1371/journal.pone.0075085)
Supplement: Table S1 — VAT prediction equations for populations of South Asian and African origin from previous studies (17, 22, 24, 26, 27, 31). (DOCX) [file pone.0075085.s002.docx]

| **Equations derived from populations of South Asian (Indian) origin** |
| --- |
| *Brundavani et el, 2008* |
| Men: VAT (cm^2^)= -382.9 + (1.09 x weight) + (6.04 x waist) – (2.29 x BMI) |
| Women: VAT (cm^2^)= -278.0 – (0.86 x weight) + (5.19 x waist) |
| *Goel et al, 2006* |
| VAT (cm^2^)= -238.7 + (16.9 x age) + (934.18 x sex) + (578.09 x BMI) - (441.06 x hip) + (434.2 x waist) |
| **Equation derived from population of African (American) origin** |
| *Stanforth et al, 2004* |
| Men: VAT (cm^2^)= exp ( -0.7 + (0.05 x BMI) + (3.20 x waist:hip ratio) + (0.02 x age) + (0.36 x race*) |
| Women: VAT (cm^2^)= exp ( 0.33 + (0.05 x BMI) + (1.85 x waist:hip ratio) + (0.02 x age) + (0.22 x race*) |
| *race: 0=African American, 1=white European |
| **Equations derived from populations of white European (North American) origin** |
| *Bonora et al, 1995* |
| *Men: VAT (cm2)= -453.7 + (6.37 x waist)* |
| Women: VAT (cm^2^)= -370.5 + (4.04 x waist) + (2.62 x age) |
| *Despres et al, 1991* |
| Men: VAT (cm^2^)= -225.39 + (2.13 x age) + (2.84 x waist) |
| *Ross et al, 1992* |
| Men: VAT (cm^2^)= -328.16 + (2.34 x age) + (363.43 x waist:hip ratio) |
