# Supplementary material for: Estimation of CT-Derived Abdominal Visceral and Subcutaneous Adipose Tissue Depots from Anthropometry in Europeans, South Asians and African Caribbeans
Source: PLoS One. 2013 Sep 17;8(9):e75085. doi: 10.1371/journal.pone.0075085 (PMC3775834; doi:10.1371/journal.pone.0075085)
Supplement: Table S2 — Full prediction models for VAT and SAT basic and expanded models, SABRE study 2008-2011. 1Basic models used the following predictors: VAT: age (years), waist circumference (cm) and SAT: age (years), height (cm), weight (kg). 2Expanded model used the following predictors for both depots: age (years), weight (kg), height (cm), waist circumference (cm), hip circumference (cm), thigh circumference (cm). (DOCX) [file pone.0075085.s003.docx]

|  | **White European** | | | | **South Asian** | | | | **African Caribbean** | | | |
| --- | --- | --- | --- | --- | --- | --- | --- | --- | --- | --- | --- | --- |
|  | **β** | **95% CI** | **p** | **Adj. R²** | **β** | **95% CI** | **p** | **Adj. R²** | **β** | **95% CI** | **p** | **Adj. R²** |
| **VAT^1^** |  |  |  |  |  |  |  |  |  |  |  |  |
| **Men** |  |  |  |  |  |  |  |  |  |  |  |  |
| Age | 1.88 | 0.87, 2.88 | <0.001 |  | 1.44 | 0.28, 2.59 | 0.02 |  | 1.14 | -1.06, 3.37 | 0.30 |  |
| Waist | 6.57 | 6.04, 7.09 | <0.001 | 0.64 | 6.67 | 6.03, 7.30 | <0.001 | 0.59 | 7.24 | 6.13, 8.33 | <0.001 | 0.71 |
| Constant | -554.67 | -643.22, -466.11 | <0.001 |  | -523.36 | -626.09, -420.62 | <0.001 |  | -598.50 | -763.92, -433.08 | <0.001 |  |
| **Women** |  |  |  |  |  |  |  |  |  |  |  |  |
| Age | 0.68 | -0.95, 2.30 | 0.41 |  | 1.76 | -0.32, 3.84 | 0.10 |  | 1.82 | 0.01, 3.61 | 0.05 |  |
| Waist | 4.66 | 3.88, 5.44 | <0.001 | 0.59 | 3.27 | 2.08, 4.45 | <0.001 | 0.35 | 3.15 | 2.29, 4.00 | <0.001 | 0.43 |
| Constant | -328.49 | -463.19, -193.78 | <0.001 |  | -276.47 | -462.69, -90.24 | 0.004 |  | -294.41 | -447.34, -141.46 | <0.001 |  |
| **VAT^2^** |  |  |  |  |  |  |  |  |  |  |  |  |
| **Men** |  |  |  |  |  |  |  |  |  |  |  |  |
| Age | 1.87 | 0.83, 2.91 | <0.001 |  | 1.18 | 0.01, 2.36 | 0.05 |  | 0.94 | -1.25, 3.12 | 0.40 |  |
| Weight | 2.80 | 1.41, 4.19 | <0.001 |  | 2.16 | 0.50, 3.81 | 0.01 |  | - | - | - |  |
| Height | -1.79 | -2.81, -0.76 | 0.001 |  | -2.35 | -3.76, -0.94 | 0.001 |  | - | - | - |  |
| Waist | 7.57 | 6.24, 8.90 | <0.001 | 0.69 | 6.56 | 5.20, 7.91 | <0.001 | 0.61 | 8.63 | 6.86, 10.40 | <0.001 | 0.73 |
| Hip | -4.91 | -6.60, -3.21 | <0.001 |  | -2.15 | -3.74, -0.55 | 0.008 |  | -3.86 | -6.92, -0.80 | 0.01 |  |
| Thigh | -2.85 | -4.91, -0.77 | 0.007 |  | -1.81 | -3.52, -0.10 | 0.04 |  | 1.47 | -0.18, 3.12 | 0.08 |  |
| Constant | 67.88 | -167.47, 303.23 | 0.57 |  | 39.24 | -259.76, 338.23 | 0.80 |  | -422.31 | -663.63, -180.99 | 0.001 |  |
| **Women** |  |  |  |  |  |  |  |  |  |  |  |  |
| Age | 0.90 | -0.69, 2.48 | 0.27 |  | 1.13 | -1.05, 3.32 | 0.30 |  | 2.20 | 0.49, 3.91 | 0.01 |  |
| Weight | 2.71 | 1.09, 4.34 | 0.001 |  | 6.28 | 3.40, 9.16 | <0.001 |  | 3.20 | 1.16, 5.25 | 0.003 |  |
| Height | - | - | - |  | -2.47 | -4.82, -0.12 | 0.04 |  | -2.42 | -4.76, -0.08 | 0.04 |  |
| Waist | 2.62 | 1.12, 4.12 | 0.001 | 0.62 | 1.85 | 0.18, 3.52 | 0.03 | 0.55 | 3.35 | 1.80, 4.88 | <0.001 | 0.56 |
| Hip | - | - | - |  | -3.71 | -6.78, -0.63 | 0.02 |  | -4.93 | -7.00, -2.87 | <0.001 |  |
| Thigh | -1.75 | -3.76, 0.25 | 0.09 |  | -4.35 | -7.58, -1.12 | 0.009 |  | - | - | - |  |
| Constant | -250.86 | -401.68, -100.05 | 0.001 |  | 475.69 | -49.00, 1000.38 | 0.08 |  | 323.39 | -145.06, 791.85 | 0.17 |  |
| **SAT^1^** |  |  |  |  |  |  |  |  |  |  |  |  |
| **Men** |  |  |  |  |  |  |  |  |  |  |  |  |
| Age | -0.34 | -1.19, 0.55 | 0.47 |  | 1.00 | 0.10, 1.90 | 0.03 |  | 1.73 | -0.17, 3.63 | 0.07 |  |
| Weight | 5.67 | 5.27, 6.07 | <0.001 | 0.69 | 6.72 | 6.24, 7.20 | <0.001 | 0.72 | 6.49 | 5.53, 7.43 | <0.001 | 0.72 |
| Height | -3.00 | -3.80, -2.18 | <0.001 |  | -4.51 | -5.43, -3.59 | <0.001 |  | -5.34 | -7.07, -3.61 | <0.001 |  |
| Constant | 290.49 | 131.46, 449.51 | <0.001 |  | 418.22 | 256.14, 580.31 | <0.001 |  | 473.94 | 140.32, 807.56 | 0.006 |  |
| **Women** |  |  |  |  |  |  |  |  |  |  |  |  |
| Age | -0.59 | -2.49, 1.30 | 0.54 |  | -2.59 | -5.35, 0.15 | 0.06 |  | 1.51 | -1.17, 4.19 | 0.27 |  |
| Weight | 7.22 | 6.37, 8.07 | <0.001 | 0.75 | 8.52 | 7.11, 9.91 | <0.001 | 0.77 | 8.54 | 7.40, 9.68 | <0.001 | 0.76 |
| Height | -4.09 | -6.14, -2.03 | <0.001 |  | -7.39 | -10.05, -4.72 | <0.001 |  | -6.50 | -9.57, -3.42 | <0.001 |  |
| Constant | 461.22 | 96.13, 826.31 | 0.01 |  | 1071.9 | 550.47, 1593.34 | <0.001 |  | 619.42 | 57.94, 1180.89 | 0.03 |  |
| **SAT^2^** |  |  |  |  |  |  |  |  |  |  |  |  |
| **Men** |  |  |  |  |  |  |  |  |  |  |  |  |
| Age | -1.19 | -2.06, -0.31 | 0.008 |  | 0.63 | -0.25, 1.51 | 0.16 |  | -0.08 | -1.74, 1.58 | 0.92 |  |
| Weight | 2.29 | 1.17, 3.40 | <0.001 |  | 3.88 | 2.76, 5.01 | <0.001 |  | - | - | - |  |
| Height | -2.00 | -2.84, -1.15 | <0.001 |  | -3.21 | -4.22, -2.18 | <0.001 |  | -2.09 | -3.50, -0.69 | 0.00 |  |
| Waist | 2.34 | 1.22, 3.45 | <0.001 | 0.72 | 1.43 | 0.43, 2.42 | 0.005 | 0.75 | 3.83 | 2.47, 5.19 | <0.001 | 0.81 |
| Hip | 2.49 | 1.08, 3.89 | 0.001 |  | 2.54 | 1.34, 3.73 | <0.001 |  | 6.52 | 4.18, 8.86 | <0.001 |  |
| Thigh | - | - | - |  | - | - | - |  | -1.23 | -2.48, 0.01 | 0.05 |  |
| Constant | -28.95 | -209,87, 151.98 | 0.75 |  | 48.58 | -154.86, 252.02 | 0.64 |  | -373.61 | -674.53, -72.70 | 0.02 |  |
| **Women** |  |  |  |  |  |  |  |  |  |  |  |  |
| Age | -0.99 | -2.71, 0.73 | 0.26 |  | -1.72 | -4.28, 0.83 | 0.18 |  | 0.23 | -1.74, 2.20 | 0.82 |  |
| Weight | 3.76 | 1.33, 6.20 | 0.003 |  | 3.94 | 0.99, 6.88 | 0.01 |  | - | - | - |  |
| Height | -3.07 | -5.05, -1.08 | 0.003 |  | -5.21 | -7.95, -2.46 | <0.001 |  | - | - | - |  |
| Waist | - | - | - | 0.80 | - | - | - | 0.81 | 2.24 | 0.75, 3.73 | 0.004 | 0.86 |
| Hip | 7.01 | 3.78, 10.24 | <0.001 |  | 5.63 | 2.36, 8.91 | 0.001 |  | 9.48 | 7.69, 11.27 | <0.001 |  |
| Thigh | -3.03 | -5.45, -1.16 | 0.003 |  | - | - | - |  | - | - | - |  |
| Constant | 24.98 | -427.98, 477.95 | 0.91 |  | 404.25 | -208.87, 1017.38 | 0.19 |  | -896.27 | -1084.19, -708.35 | <0.001 |  |
