# Supplementary material for: Estimation of CT-Derived Abdominal Visceral and Subcutaneous Adipose Tissue Depots from Anthropometry in Europeans, South Asians and African Caribbeans
Source: PLoS One. 2013 Sep 17;8(9):e75085. doi: 10.1371/journal.pone.0075085 (PMC3775834; doi:10.1371/journal.pone.0075085)
Supplement: Table S3 — Sensitivity analyses of VAT and SAT prediction models: effects on adjusted R2. Empty cells indicate when terms did not stay in the model after backwards stepwise regression. (DOCX) [file pone.0075085.s004.docx]

|  |  | **Men** |  |  | **Women** |  |
| --- | --- | --- | --- | --- | --- | --- |
| **Model** | **White** | **South** | **African** | **White** | **South** | **African** |
|  | **European** | **Asian** | **Caribbean** | **European** | **Asian** | **Caribbean** |
| **VAT** |  |  |  |  |  |  |
| **Base model: age, waist circumference** | 0.64 | 0.59 | 0.71 | 0.59 | 0.35 | 0.43 |
| **Base model + BMI, kg/m²** | 0.64 | 0.59 | - | 0.60 | - | - |
| **Base model + total body fat mass, kg + fat %** | - | 0.61 | 0.74 | 0.64 | - | 0.45 |
| **Base model in participants with diabetes** | 0.66 | 0.59 | 0.76 | 0.71 | 0.64 | 0.5 |
| **Base model in participants without diabetes** | 0.63 | 0.59 | 0.69 | 0.59 | 0.27 | 0.39 |
| **Base model in participants aged 56-72** | 0.65 | 0.59 | 0.77 | 0.66 | 0.56 | 0.35 |
| **Base model in participants aged 73-86** | 0.64 | 0.66 | 0.61 | 0.54 | 0.26 | 0.62 |
| **SAT** |  |  |  |  |  |  |
| **Base model: age, weight, height** | 0.69 | 0.72 | 0.72 | 0.75 | 0.77 | 0.76 |
| **Base model + BMI, kg/m²** | - | 0.72 | 0.74 | - | 0.79 | - |
| **Base model + total body fat mass, kg + fat %** | 0.72 | 0.76 | 0.78 | 0.76 | 0.80 | 0.74 |
| **Base model in participants with diabetes** | 0.65 | 0.77 | 0.63 | 0.59 | 0.69 | 0.55 |
| **Base model in participants without diabetes** | 0.72 | 0.69 | 0.85 | 0.81 | 0.79 | 0.86 |
| **Base model in participants aged 56-72** | 0.74 | 0.75 | 0.79 | 0.76 | 0.78 | 0.75 |
| **Base model in participants aged 73-86** | 0.67 | 0.68 | 0.70 | 0.80 | 0.63 | 0.81 |
